# Implications of post-translational modifications of IRF7 on pDC IFN-alpha response

**DOI:** 10.1186/1742-4690-9-S2-P176

**Published:** 2012-09-13

**Authors:** M Griesbeck, E Doyle, RJ Lindsay, J Boucau, S LeGall, M Altfeld, JJ Chang

**Affiliations:** 1 Ragon Institute of MGH, MIT and Harvard, Charlestown, MA, USA

## Background

We previously showed that plasmacytoid dendritic cells (pDCs) derived from females can produce significantly more IFN-alpha in response to HIV-1 and HIV-1-encoded TLR7/8 ligands than pDCs derived from males, resulting in stronger secondary activation of CD8+ T cells (Meier et al., Nat Med 2009). Given the crucial role of interferon regulatory factor 7 (IRF7) in the regulation of type I IFN production by pDCs, the goal of the current study was to investigate its impact on the observed differences.

## Methods

Fresh PBMC were isolated from HIV-1-negative subjects enrolled at Massachusetts General Hospital and stimulated by either CL097 (synthetic TLR7 ligand) or AT-2 inactivated HIV-1. Phosphorylation levels of proteins involved in the TLR7 pathway including IRF7 were measured in pDCs by phospho-flow cytometry at baseline and at different time-points after TLR7 stimulation. The kinetics of IRF7 modifications in the TLR7 pathway were confirmed using mRNA expression of IFN-alpha.

## Results

Baseline levels of phosphorylated IRF7 were found to be similar between males and females. However we observed faster phosphorylation kinetics of IRF7 in females than in males using flow cytometry with phospho-IRF7 peaking in females at 20min post-stimulation and males at 30min.

## Conclusion

These data indicate that sex differences in the kinetics of IRF7 phosphorylation might account for described higher IFN-alpha production upon TLR7 stimulation in females, providing new insights into the mechanisms underlying faster HIV-1 disease progression in females compared to males after controlling for viral load (Farzadegan et al., Lancet 1998).

